# Mesenchymal stem cells conditioned with glucose depletion augments their ability to repair-infarcted myocardium

**DOI:** 10.1111/j.1582-4934.2012.01568.x

**Published:** 2012-09-26

**Authors:** Mahmood S Choudhery, Mohsin Khan, Ruhma Mahmood, Sadia Mohsin, Shoaib Akhtar, Fatima Ali, Shaheen N Khan, Sheikh Riazuddin

**Affiliations:** National Center of Excellence in Molecular Biology, University of the PunjabLahore, Pakistan

**Keywords:** MSCs, stem cell ageing, senescent heart, caloric restriction, pre-conditioning

## Abstract

Mesenchymal stem cells (MSCs) are an attractive candidate for autologous cell therapy, but their ability to repair damaged myocardium is severely compromised with advanced age. Development of viable autologous cell therapy for treatment of heart failure in the elderly requires the need to address MSC ageing. In this study, MSCs from young (2 months) and aged (24 months) C57BL/6 mice were characterized for gene expression of *IGF-1,* *FGF-2,* *VEGF,* *SIRT-1,* *AKT,* *p16^INK4a^,* *p21* and *p53* along with measurements of population doubling (PD), superoxide dismutase (SOD) activity and apoptosis. Aged MSCs displayed senescent features compared with cells isolated from young animals and therefore were pre-conditioned with glucose depletion to enhance age affected function. Pre-conditioning of aged MSCs led to an increase in expression of *IGF-1,* *AKT* and *SIRT-1* concomitant with enhanced viability, proliferation and delayed senescence. To determine the myocardial repair capability of pre-conditioned aged MSCs, myocardial infarction (MI) was induced in 24 months old C57BL/6 wild type mice and GFP expressing untreated and pre-conditioned aged MSCs were transplanted. Hearts transplanted with pre-conditioned aged MSCs showed increased expression of paracrine factors, such as *IGF-1,* *FGF-2,* *VEGF* and *SDF-1α*. This was associated with significantly improved cardiac performance as measured by d*p*/d*t*_max_, d*p*/d*t*_min_, LVEDP and LVDP, declined left ventricle (LV) fibrosis and apoptosis as measured by Masson's Trichrome and TUNEL assays, respectively, after 30 days of transplantation. In conclusion, pre-conditioning of aged MSCs with glucose depletion can enhance proliferation, delay senescence and restore the ability of aged cells to repair senescent infarcted myocardium.

## Introduction

Heart failure is the leading cause of death in a large segment of population with advanced age. Mesenchymal stem cells (MSCs) derived from the bone marrow represent an attractive source for cell based interventional approaches to treat heart damage because of their ability to support angiogenesis [1–3], form functionally competent cardiomyocytes [4, 5] and enhance progenitor cell recruitment [6]. However, stem cell ability to re-populate the heart and support myocardial repair declines with advanced age [7] representing a severe limitation in the development of an autologous cell delivery option for aged patients with myocardial infarction.

Pre-conditioning strategies aimed at limiting age related effects on stem cells have recently been employed with success. These include treatment with cyclin dependent kinase inhibitors [8], exposure to low temperature [9] and overexpression of antioxidant genes like sirtuins and heat–shock proteins [10]. However, some available techniques for pre-conditioning may increase chances of cancer development [11], whereas others are preferentially effective for young cells rather than aged cells [9]. Recent *in vivo* studies using caloric restriction demonstrate beneficial effects on longevity of organisms ranging from yeast to primates [12]. In particular, caloric restriction has a significant effect on destructive cellular processes, such as oxidative and glycation damage, and thus can slow down ageing and cell death [9, 13, 14]. Similarly, varying glucose levels in cellular microenvironment has remarkable effects on apoptosis, senescence, differentiation and proliferation of mesenchymal stem cells [15]. Therefore, caloric restriction has emerged as an experimental intervention that can safely and reproducibly enhance life span, nevertheless, its effect on stem cell ageing and function remains largely unexplored.

MSC ability to augment myocardial repair, following injury declines with age [16] meriting the requirement for a strategy aimed to increase MSC reparability. Therefore, we hypothesized that pre-conditioning of aged MSCs would enhance their ability to repair myocardium after infarction. We have demonstrated in this study that pre-conditioning of aged MSCs with glucose depletion can significantly enhance viability, proliferation and survival signalling. Furthermore, transplantation of the pre-conditioned aged MSCs in senescent heart with MI resulted in improved cardiac performance as compared with aged untreated MSCs.

## Materials and methods

### Animals

In this study young (2 months) and aged (24 months) mice were used. The animals were kept and maintained in the animal house facility of National Center of Excellence in Molecular Biology, University of the Punjab, according to the procedures approved by the institutional committee for the care of animals.

### Cell culture

MSCs were isolated from tibias and femora of 2 and 24 months old C57BL/6 mice, according to their ability to adhere to plastic surface of a culture flask and cultured as described previously [16]. In addition for transplantation, MSCs were isolated from 24 months old C57BL/6 transgenic green fluorescence protein (GFP) expressing mice.

### Growth kinetics

MSCs were serially subcultured under standard conditions for analysis of PD. Briefly, at first passage, 1 × 10^5^ cells were counted and plated in a 25 cm^2^ culture flask. At 90% confluency, cells were subcultured by counting and plating at the same density as described above. This procedure was repeated until the cells were unable to reach 90% confluency even after 4 weeks [9]. Number of PDs between passages were determined by using the formula: No. of PDs = Log10 (*N*/*N*_0_) × 3.33, where PDs represents population doublings, *N* is the number of cells when harvested and *N*_0_ is number of cells seeded.

### Immunocytochemistry

MSCs from young and aged animals were characterized for expression of various stem cell markers. The primary antibodies used were rat monoclonal anti-CD44 (BD Pharmingen, San Diego, CA, USA), mouse monoclonal anti-CD45 (Santa Cruz, CA, USA), mouse monoclonal anti-CD90 (Abcam, Cambridge, MA, USA), rabbit polyclonal anti-CD105 (Abcam), mouse monoclonal anti-p16 (Santa Cruz) and rabbit polyclonal anti p53 (Santa Cruz). Secondary antibodies used were donkey anti-rat IgG, anti-mouse IgG, anti-rabbit IgG (Jackson Immunoresearch, West Grove, PA, USA) conjugated with either rhodamine or FITC. Nuclei were stained with 4, 6-diamidino-2-phenylindole (DAPI) and images were captured using a BX-61 microscope equipped with DP-70 digital camera (Olympus, Tokyo, Japan). Furthermore, MSCs from young and aged animals were stained with FITC labelled Annexin-V Kit (Abcam) to measure apoptotic cells after exposure to oxidative stress induced by H_2_O_2_.

### Superoxide dismutase activity

Superoxide dismutase activity was determined in MSCs from young and aged animals by SOD activity colorimetric assay kit (Abcam) according to the manufacturer's protocol. Briefly, protein was isolated using lysis buffer from both sets of MSCs and SOD activity was measured using 10 μg of the total protein extract. Absorbance values were measured by using Spectra max PLUS 384 (Molecular Devices, Sunnyvale, CA, USA) at 450 nm.

### Pre-conditioning of aged MSCs

Aged MSCs were pre-conditioned with serum free Dulbecco's modified eagle medium lacking glucose (GsF-M) for 1, 6 and 12 hrs. Medium without serum and with normal glucose concentration (4.5 g/l, NsF-M) was used as control at similar time-points. MSCs were recovered in normal medium for 1 hr prior to RNA extraction, viability, proliferation, senescence assays and *in vivo* transplantation experiments.

### Immunoblot

Immunoblot analysis was performed to measure the expression of *pAKT* in aged MSCs and aged pre-conditioned MSCs. Protein was extracted using RIPA buffer and loaded into each well of a 10% polyacrylamide gel. The electrophoresed proteins were then transferred to nitrocellulose membrane (Amersham, Piscataway, NJ, USA) and incubated with 5% skim milk in Tris-buffer for 1 hr on the shaker. The membranes were then incubated against anti-p-AKT473 (Santa Cruz) overnight at 4°C. After washing, the membranes were incubated with HRP-conjugated secondary antibodies for 1 hr on shaker, washed and developed with DAB substrate kit (Zymed laboratories Inc., San Francisco, CA, USA).

### Gene expression profiling of MSCs

RNA was extracted from aged control and aged pre-conditioned MSCs with trizole reagent (Invitrogen Corporation, Grand Island, NY, USA) and quantified with ND-1000 spectrophotometer (NanoDrop Technologies, Wilmington, DE, USA). cDNA synthesis was carried out from 1 μg of RNA sample with M-MLV reverse transcriptase (Invitrogen Corporation). RT–PCR analysis for *IGF-1, VEGF, FGF-2, SIRT-1, AKT, p53, p16*^*INK4a*^*, p21, PCNA, BCL-2, BCL-xL, BAX and BAK* was carried out using a GeneAmp PCR system 9700 (Applied Biosystem, Carlsbad, CA, USA) with *GAPDH* as internal control. Gels were quantified using Quantity One® 1-D Analysis Software, version 4.4 (Bio-Rad Laboratories Inc., Hercules, CA, USA) according to the instructions given in user's guide, with quantity of the ladder (Fermentas International Inc., Glen Burnie, MD, USA) used as standard. The sequences (5′-3′) for the primer pairs and their product lengths (bp) have been mentioned in Table 1.

**Table 1:** Primer sequences

Gene	Primer sequence	Size (bp)
IGF-1	AGGCTATGGCTCCAGCATTC (F)	166
	AGTCTTGGGCATGTCAGTGTG (R)	
VEGF	ACCCCGACGAGATAGAGTACAT (F)	200
	CTTCTAATGCCCCTCCTTGT (R)	
SDF-1	CGGCTGAAGAACAACAACAG (F)	122
	GGGGGTCTACTGGAAAGTCC (R)	
FGF-2	TGTCTATCAAGGGAGTGTGTGC (F)	154
	CAACTGGAGTATTTCCGTGACC (R)	
AKT	CCTCAAGAACGATGGCACCT (F)	151
	CAGGCAGCGGATGATAAAGG (R)	
SIRT-1	TAGGGACCTTTGCCTCATC (F)	100
	GGCATTCACCACCTAGCC (R)	
p53	AGCATCTTATCCGGGTGGAAG (F)	157
	CCCATGCAGGAGCTATTACACA (R)	
p21	GTACTTCCTCTGCCCTGCTG (F)	171
	AGAAGACCAATCTGCGCTTG (R)	
Bax	TGGAGATGAACTGGACAGCA (F)	152
	CAAAGTAGAAGAGGGCAACCAC (R)	
Bak	CAGGACACAGAGGAGGTCTTTC	182
	TAGCGCCGGTTAATATCATCTC	
Bcl-2	GATGACTTCTCTCGTCGCTACC	182
	ACGCTCTCCACACACATGAC	
p16^INK4a^	GCTCAACTACGGTGCAGATTC (F)	196
	TCGCACGATGTCTTGATGTC (R)	
PCNA	GGTTGGTAGTTGTCGCTGTA (F)	720
	CAGGCTCATTCATCTCTATCG (R)	
GATA-4	ACCCATAGTCACCAAGGCTG (F)	197
	ACCCATAGTCACCAAGGCTG (R)	
MEF-2	CACGCCTGTCACCTAACATCC	178
	TGTTAGGTCTCAAACGCCAC	
NKx2.5	GGTCTCAATGCCTATGGCTAC (F)	142
	GTTCACGAAGTTGCTGTTGG (R)	
GAPDH	CTCTTGCTCTCAGTATCCTTG (F)	370
	GCTCACTGGCATGGCCTTCCG (R)	

### Senescence

Cellular senescence was measured in untreated control and pre-conditioned aged MSCs with Senescence Detection Kit (Abcam) according to manufacturer's instructions. Briefly, aged MSCs pre-conditioned with GsF-M for 1 hr were cultured in NsF-M with 15% FBS for up to 24 days. The cells were stained with senescence associated β-galactosidase solution at 8, 16 and 24 days of pre-conditioning. Fluorescent images of treated and control groups were taken with Olympus BX-61 microscope equipped with DP-70 digital camera (Olympus).

### Real time RT-PCR

RNA was extracted from the hearts of all groups with Trizol reagent (Invitrogen, Corporation) and quantified with ND-1000 spectrophotometer (NanoDrop Technologies). One microgram of RNA was used for cDNA synthesis with M-MLV reverse transcriptase (Invitrogen Corporation). Real Time RT–PCR analysis for *GATA-4, MEF-2* and *NKX2.5* was carried out with SYBR Green PCR Super Mix (Bio-Rad Lab) on BioRad system iQ5 as described earlier [17]. The sequences (5′-3′) for the primer pairs and their product lengths (bp) have been mentioned in Table 1.

### Proliferation

Cell proliferation assay was performed with XTT (sodium 3–[1-{phenylaminocarbonyl}-3,4-tetrazolium]-bis {4-methoxy-6-nitro} benzene sulphonic acid hydrate) according to the manufacturers’ instructions (Roche, Indianapolis, IN, USA). Briefly, 4 × 10³ aged pre-conditioned and untreated MSCs were plated in a flat bottom 96-well plate. Cell proliferation was measured after 1, 6 and 12 hrs of pre-conditioning treatment. Absorbance values were measured with Spectra max PLUS 384 (Molecular Devices) at 450 nm with 650 nm as reference wavelength.

### Cell viability

Viability in aged pre-conditioned and untreated MSCs was measured with viability assay kit (Abcam) according to the manufacturer's instructions. Briefly, both types of aged MSCs were plated in triplicate in a flat bottom 96-well plate, pre-conditioned as described above and then viability was measured for 1, 6 and 12 hrs. Fold increase in ATP levels was noted in a luminometer (Modulus Microplate Luminometer; Turner Biosystem, Sunnyvale, CA, USA).

### Myocardial infarction and cell transplantation

Myocardial infarction was produced in wild type C57BL/6 aged mice (24 months old) under anaesthesia with sodium pentobarbital (40 mg/kg) as described previously [16]. In brief, heart was exposed by left side limited thoracotomy and LAD was ligated with 7–0 silk suture 1 mm from the tip of the normally positioned left auricle. Animals with MI were divided into three groups (*n* = 16) each. Group I mice were injected with normal saline and represented the control group. Groups II and III mice were transplanted with aged MSCs and aged pre-conditioned MSCs, respectively, in the border of the infarct area. MSC concentration used in the experiments was 1 × 10^6^/ml and animals were given intramyocardial injections of MSCs (20 μl–100,000 cells/animal) at the time of LAD ligation.

### Assessment of heart function

Mice were anaesthetized with sodium pentobarbital (40 mg/kg) by intraperitoneal injection and cardiac function was assessed with Millar Apparatus as described previously [16]. Briefly, hemodynamic parameters were recorded with a micro tip pressure transducer catheter (SPR-839; Millar Instrument, Houston, TX, USA) connected to MPCU-400 *P–V* signal conditioning hardware for data acquisition. LV systolic function was evaluated by *P*_max_, d*p*/d*t*_max_ and arterial elastance. LV diastolic function was evaluated by end-diastolic pressure and d*p*/d*t*_min_. Hemodynamic parameter analysis was carried out using Millar's PVAN software (Version 3.3).

### Measurement of fibrosis and apoptosis

Fibrosis and apoptosis were measured in hearts transplanted with aged MSCs by using Masson's Trichrome and TUNEL assay as previously described [16].

### Immunohistochemistry

Mice were killed 30 days after MSC transplantation. The hearts were fixed with 4% paraformaldehyde and snap frozen in liquid nitrogen. Sections were cut at 5–8 μm thick in a cryostat (Microme, Walldorf, Germany) at −20°C. Sections were stained with anti-CD31 (Chemicon, Temecula, CA, USA) to evaluate angiogenesis. The secondary antibody used was donkey antimouse conjugated with FITC. Capillary density was measured by identifying CD31 expressing capillaries and small arterioles per five random fields within two separate sections of each group.

### Statistical analysis

Quantitative data were obtained from two coverslips each from three separate experiments for MSC characterization. Five random fields per cover slip were analysed and the data were expressed as mean ± S.E.M. Hemodynamic parameters were assessed by one-way analysis of variance anova (*P*-value of <0.05 was considered statistically significant). Analysis of viability, proliferation, β-gal associated senescence, percentage of fibrosis, comparison of vascular density and measurements of apoptotic nuclei between groups was performed by Student's unpaired *t*-test (*P*-value of <0.05 was considered statistically significant).

## Results

### *In vitro* studies

#### Characterization of young and aged MSCs

**Fig 1:**
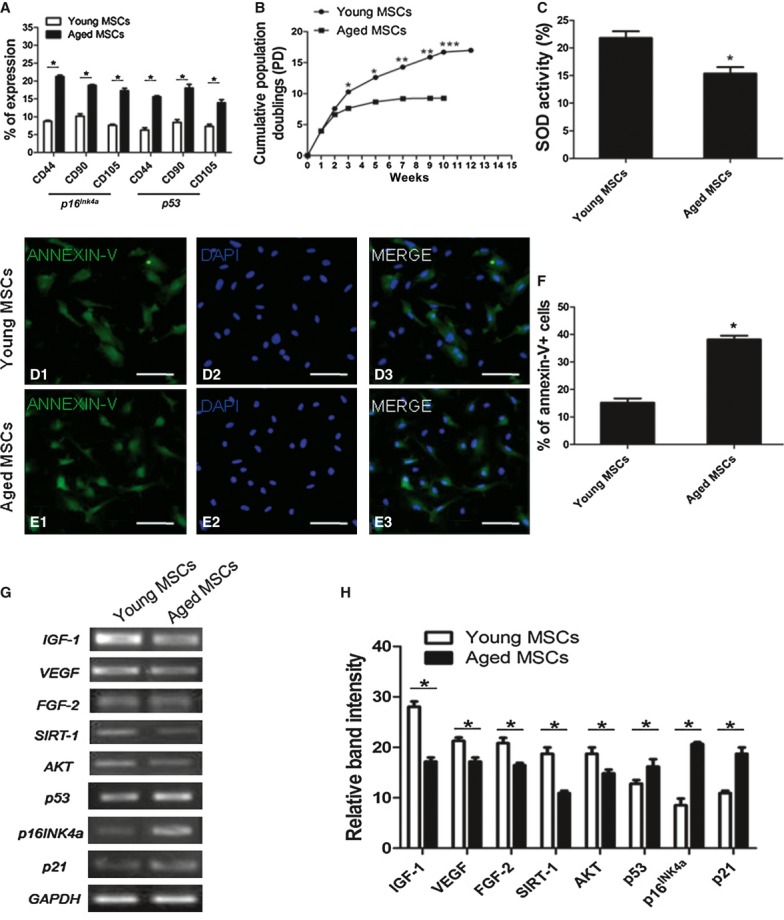
Characterization of young and aged MSCs. (A) Aged MSCs have higher expression of *p53* and *p16*^*INK4a*^ as indicated by double immunostaining with MSCs markers and with either *p16*^*INK4a*^ or *p53*. (B) Growth kinetics of mesenchymal stem cells showing higher number of cumulative population doublings of young MSCs than aged MSCs. (C) Higher percentage of superoxide dismutase activity in young as compared with aged MSCs. (D and E) Measurement of apoptosis in young and aged MSCs. (D1, D2 and D3) Young MSCs. (E1, E2 and E3) Aged MSCs. (F) Bar graph showing percentage of Annexin-V positive cells in young and aged MSCs. (G) Gene profiling of young and aged MSCs. (H) Gel quantification of all genes using Image J Software. *P < 0.05, **P < 0.01, ***P < 0.001 for young MSCs *versus* aged MSCs.

The young and aged MSCs were characterized for the expression of stem cell markers CD44, CD45, CD90 and CD105 (Fig. 1A). Co-labelling of MSCs with p16^INK4a^ and p53 showed an increased expression in MSCs from aged animals compared with young animals. Concurrently, PD analysis of young and aged MSCs showed that aged cells have significantly decreased life span compared with young cells (Fig. 1B). SOD activity was significantly increased in young MSCs compared with aged cells indicating superior antioxidant defence mechanisms in young MSCs (Fig. 1C). Furthermore, annexin-V staining showed increased expression of apoptotic cells in aged MSCs compared with young cells (Fig. 1D–F). Gene expression profile of young and aged MSCs showed higher levels of *IGF-1, VEGF, FGF-2, SIRT-1, AKT* in contrast to lower levels of *p53, p16*^*INK4a*^ and *p21* in young MSCs compared with aged cells (Fig. 1G and H).

#### Pre-conditioning of aged MSCs

**Fig 2:**
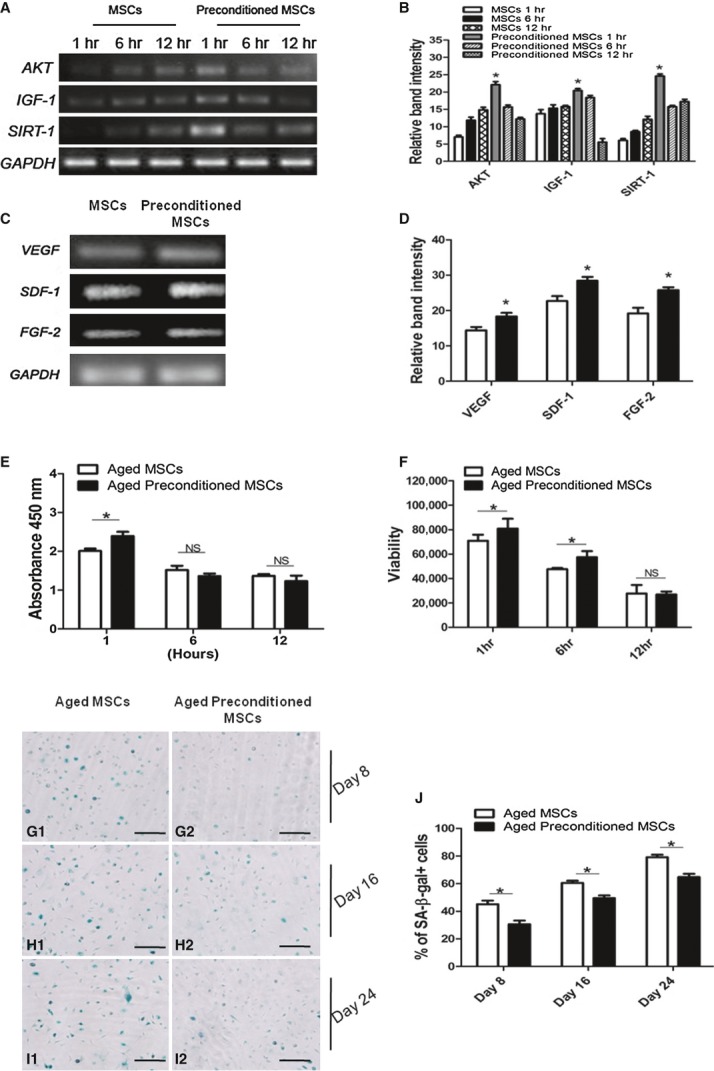
Pre-conditioning of Aged MSCs. (A) RT-PCR analysis for the expression of AKT, IGF-1 and SIRT-1 in aged control and pre-treated MSCs. (B) Gel quantification of RT-PCR bands. (C) Gene expression of *VEGF*, *SDF-1* and *FGF-2* after 1 hr of pre-conditioning. (D) Gel quantification of all genes using Image J software. (E) Effect of caloric restriction on cell proliferation of aged MSCs. Absorbance was measured at 450nm and was higher in 1 hr pre-treated group. (F) Cell viability assay performed in a luminometer. The results showed a significant increase in viability of aged MSCs after 1 and 6hr pre-treatment compared with untreated aged MSCs. (G–I) Senescence associated β-galactosidase staining after 8, 16 and 24 days. (G1 and G2) after 8 days, (H1 and H2) after 16 hrs, (I1 and I2) after 24 hrs. (J) Percentage expression of senescence associated β-gal. The number of β-gal positive cells was significantly higher in aged untreated MSCs than aged pre-treated MSCs. The values are expressed in mean ± SEM. *P < 0.05, **P < 0.01, ***P < 0.001 for aged MSCs *versus* aged pre-conditioned MSCs.

*Gene expression:* Aged MSCs pre-conditioned with glucose free medium lacking serum for 1, 6 and 12 hrs showed up-regulation of pro-survival genes *AKT, IGF-1* and *SIRT-1* compared with control cells treated with normal glucose, but without serum medium (Fig. 2A and B). However, pre-conditioning effect was significant 1 hr after treatment and therefore was selected as the pre-conditioning strategy for further assays. Similarly, phosphorylation of AKT was significantly higher in pre-conditioned aged MSCs compared with aged non-treated cells after 1 hr (Supplementary Figure 1A and B). Gene expression of paracrine factors, such as *VEGF, SDF-1* and *FGF-2* were observed to significantly higher in aged pre-conditioned MSCs after 1 hr of treatment compared with the non-treated aged MSCs (Fig. 2C and D).

*Proliferation and viability:* Aged MSCs were treated with caloric restricted medium for 1, 6 and 12 hrs and proliferation was measured by with commercially available XTT proliferation assay kit (Fig. 2E). The maximum proliferative response after pre-conditioning of aged MSCs with caloric restricted medium was observed at 1 hr. Whereas in 6 and 12 hrs groups the difference was not significant. Cell viability as determine by ATP levels also showed significant increase after 1 hr glucose free pre-conditioning (Fig. 2F). PD was analysed after pre-conditioning of aged MSCs in short-term culture and found to be significantly increased than non-treated aged MSCs (data not shown).

*Senescence:* Pre-conditioning delayed the onset of senescence at least for 24 days compared with control groups as measured by β-gal associated senescence (Fig. 2G–J). The difference in senescence between control and treated groups was increased gradually from day 4 onwards and was highest at day 16 and then decreased time dependently up to day 24.

### *In vivo* studies

#### Hemodynamic parameters

**Fig 3:**
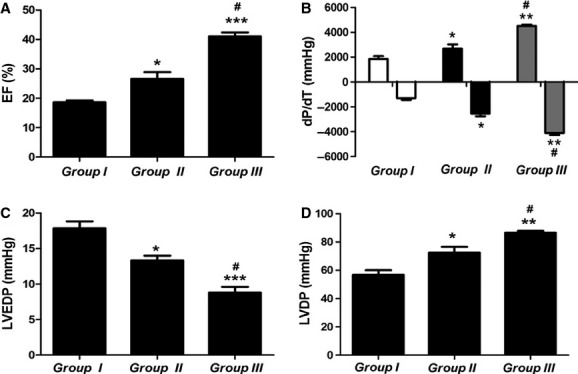
Assessment of cardiac function 30 days after stem cell transplantation. (A) Ejection fraction (%) in three groups. (B) Maximal rates of pressure decrease and increase. (C) Left ventricle end diastolic pressure. (D) Left ventricle diastolic pressure. *P < 0.05, **P < 0.01, ***P < 0.001 *versus* group I: #*P* < 0.05, ##*P* < 0.01, ###*P* < 0.001 *versus* group II.

The cardiac function of the mice transplanted with aged pre-conditioned or untreated MSCs was assessed with Millar's Apparatus, 30 days after transplantation of MSCs (Fig. 3A–D). Decreased contractility in group I mice was observed compared with group II and group III, as indicated by shifting of *P*–*V* loops to the right and also by a decrease in the amplitude of d*p*/d*t*_max_ and d*p*/d*t*_min_. Similarly, the LV diastolic function as indicated by Ped was significantly improved after transplantation of MSCs. Overall significantly increased hemodynamic parameters were observed in group III compared with group II.

#### Fibrosis

**Fig 4:**
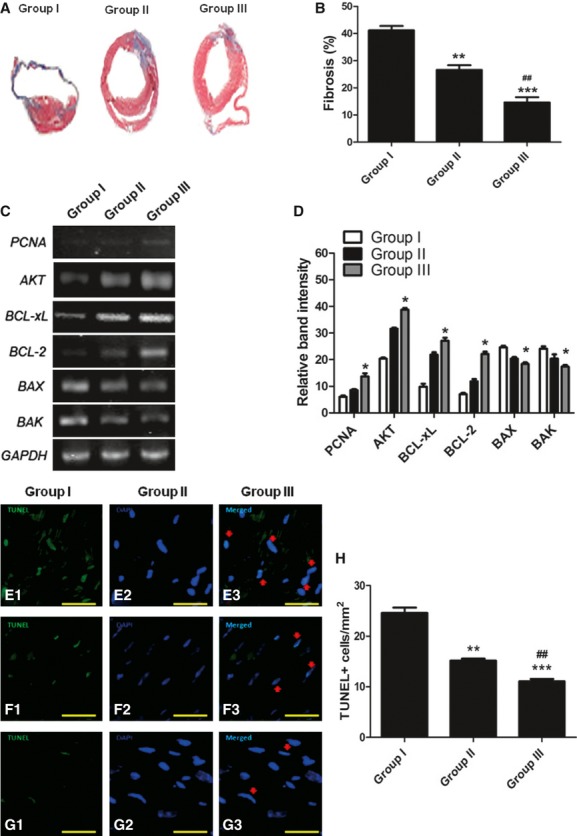
Reduced fibrosis and apoptosis in the senescent heart after transplantation of pre-conditioned aged MSCs. (A) Masson's trichrome staining of heart sections 30 days after transplantation showing fibrosis in group I, group II and group III. Fibrosis was highest in group I. (B) Bar graph indicates the percentage of fibrosis in left ventricle in aged mice. *P < 0.05, **P < 0.01, ***P < 0.001 *versus* group I: #*P* < 0.05, ##*P* < 0.01, ###*P* < 0.001 *versus* group II. (C) Expression of pro-survival and pro-apoptotic genes. Increase in expression of pro-survival genes and decrease in pro-apoptotic genes was detected after transplantation of MSCs. (D) Quantification of gel bands with Image J software. (E) TUNEL assay staining of mouse infarcted hearts for the identification of DNA fragmentation in myocytes. A statistical difference was observed in the apoptotic index among three groups. (F) Analysis of apoptotic myocyte nuclei with TUNEL Assay. *P < 0.05, **P < 0.01, ***P < 0.001 *versus* group I: #*P* < 0.05, ##*P* < 0.01, ###*P* < 0.001 *versus* group II.

Heart sections from all the groups were stained with Masson's Trichrome to measure the level of fibrosis. A considerable decrease in fibrotic area after transplantation of MSCs was observed in group III compared with group II. In control group extensive fibrosis was observed as evidenced by thinning of left ventricular wall, whereas transplantation of MSCs significantly reduced fibrosis in the myocardium (Fig. 4A and B).

The percentage of fibrosis in group II and Group III was 22.38 ± 1.94 and 14.47 ± 0.36, respectively, thus showing marked reduction compared with group I which was 51.01 ± 3.17. However, percentage of fibrosis was significantly reduced in group III mice transplanted with pre-conditioned aged MSCs compared with untreated aged MSC group.

#### Gene expression in the senescent heart

Expression of pro-survival genes *PCNA* (proliferating cell nuclear antigen), *BCL-2, BCL-xL* and *AKT-1* was up-regulated 7 days after transplantation in both group II and group III compared with group I, but significantly higher in group III. Conversely, expression of pro-apoptotic genes *BAX* and *BAK* was higher in group I compared with group II and group III, but significantly lower in group III compared with group II (Fig. 4C and D).

**Fig 5:**
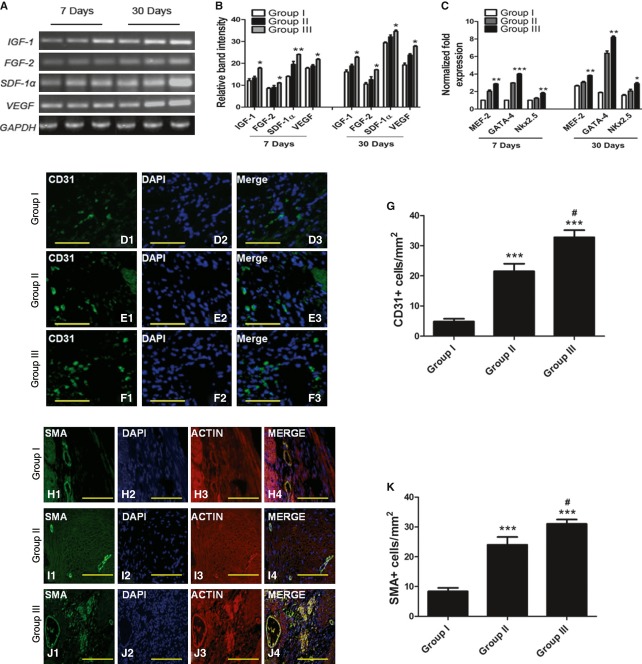
Enhanced paracrine signalling, angiogenesis and cardiac transcription factors in senescent hearts after transplantation of pre-conditioned aged MSCs. (A) RT-PCR shows increased expression of *IGF-1,* *FGF-2,* *VEGF* and *SDF-1α* 7 and 30 days after transplantation in group III hearts. (B) Quantification of gel bands with Image J software. (C) Increased expression of cardiac transcription factors in senescent hearts of group III animals, 7 and 30 days after transplantation as measured by qRT-PCR. (D–F) Blood vessel density analysis by fluorescent immunostaining for the expression of endothelial marker CD31 in all three groups. Nuclei were stained with DAPI. (G) Quantification of CD31 expression in all three groups. (H–J) SMA expression in group I, II and III transplanted hearts. (K) Percentage blood vessel density as measured by SMA expression in all three groups. *P < 0.05, **P < 0.01, ***P < 0.001 *versus* group I: #*P* < 0.05, ##*P* < 0.01, ###*P* < 0.001 *versus* group II.

It has been shown that MSCs transplanted into the ischaemic heart mediate their effect by release of cytokines that exert beneficial effects on the surrounding cells [1, 18]. RT-PCR analysis of heart showed increased expression level of *IGF-1, FGF-2, VEGF* and *SDF-1α* in group II and group III compared with group I, 30 days after transplantation. However, the expression of these growth factors was higher in group III compared with group II (Fig. 5A and B).

#### Apoptosis in the senescent heart

Level of apoptosis within the infarcted myocardium after transplantation of MSCs was measured by TUNEL assay 7 days after MI in group I, group II and group III. The number of TUNEL positive nuclei reduced in group III (11.07 ± 0.47) and group II (15.15 ± 0.41) compared with group I (34.59 ± 1.05). However, reduction in apoptosis in group III hearts transplanted with pre-conditioned aged MSCs was significantly lower compared with group II (Fig. 4E–H).

#### Expression cardiac transcription factors in senescent heart

The expression of cardiac transcription factors like *MEF-2, NKX2.5* and *GATA-4* is very important for the transdifferentiation of MSCs into cardiomyocytes [19]. Differentiation of aged MSCs into cardiac cells was confirmed 7 and 30 days after transplantation of MSCs by real time RT-PCR analysis of all groups. The expression level of *MEF-2, NKX2.5* and *GATA-4* was significantly higher in group II and group III as compared with group I. When group II and Group III were compared, the results showed higher expression of *MEF-2, NKX2.5* and *GATA-4* in group III compared with group I (Fig. 5C).

#### Angiogenesis in the senescent heart

Angiogenesis was assessed with CD31 and smooth muscle actin (α-SMA), 30 days after transplantation of MSCs. The results showed a significantly higher expression of CD31 (Fig. 5D–G) and SMA (Fig. 5H–K) in hearts transplanted with MSCs (group II and group III) compared with control (group I). However, the expression of CD31 and SMA was markedly high in group III as compared with group II (Fig. 5G and K).

## Discussion

Advance age is a major risk factor for myocardial infarction, highlighted by diminished repair and differentiation potential of stem cells limiting their potency for myocardial repair [20, 21]. Recent successes of adoptively transferred MSCs for treatment of MI have labelled them as preferred stem cell type. Autologous stem-cell therapy seems like the most feasible option to treat cardiovascular diseases, however, significant improvements in the ability of aged cells to repair and survive in the senescent heart are necessary. Therefore, we hypothesized to pre-condition aged MSCs with glucose depletion to enhance their ability to repair senescent heart. The main findings of our study are: (i) Donor age has negative impact on MSC function, (ii) *In vitro* pre-conditioning with glucose depletion improves age depleted function of MSCs and (iii) Functional improvement of senescent heart after transplantation of pre-conditioned aged MSCs.

MSCs from GFP expressing young and aged animals characterized for stem cell markers (CD44, CD90, CD105 and CD45) were co-labelled either with p16^INK4a^ or p53. Recent evidence suggests p16^INK4a^ and p53 as markers of cellular senescence and apoptosis, respectively [22, 23], therefore we sought to examine age related expression of these markers in MSCs. A significant increase in expression of p16^INK4a^ and p53 in aged MSCs was observed compared with their younger counterparts suggesting a possible role of these proteins in functional impairment of stem cells with age. Increased apoptosis was observed in aged MSCs after hydrogen peroxide induced oxidative stress and was concurrent with low SOD activity. In comparison, young MSCs displayed high levels of SOD activity and significantly low apoptosis. These findings are in agreement with reports demonstrating increased ROS levels in stem cells to be associated with senescent growth arrest, loss of self renewal and ageing [24–26]. In addition, we showed that aged MSCs display impaired growth kinetics compared with young cells as measured by PD analysis and are in concordance with previous studies [27].

In an attempt to resurrect the age depleted function of MSCs, we hypothesized that glucose deprivation can increase function of aged MSCs. Our results showed that 1 hr pre-conditioning with GsF-M increases viability, proliferation and protective signalling in aged MSCs. These findings concur with *in vivo* studies showing caloric restriction (CR) to extend mean and maximal life span in various species including rats, mice, fish, flies, worms and yeast [28]. CR has been shown to retard ageing *in vivo* and recently was shown to exert a protective effect on mouse and rat hearts [29–31]. Whereas, CR has been shown to maintain stem-like state, stem cell pool and enhance survival of cardiac stem cells within the heart [32–34], high glucose can lead to senescent changes in MSCs leading to functional impairment [9]. High glucose can create a pro-apoptotic milieu that reduces functionality and regenerative capacity of MSCs [15]. We measured senescence associated β-gal activity in aged MSCs after pre-conditioning to determine whether glucose depletion can retard ageing. Results indicate a significantly high β-gal activity in untreated MSCs compared with pre-conditioned cells with peak difference after 12 days and persisting for even 24 days after treatment. These results are in accordance with previous studies that demonstrate caloric restriction can delay senescence in T cells [35].

One important aspect of the study was to demonstrate whether aged MSCs pre-conditioned with glucose depletion can augment senescent heart function after myocardial infarction. Our results showed that aged MSCs treated with glucose depletion can improve function of the senescent heart compared with untreated cells. A possible explanation for improved heart function may stem from an enhanced ability of pre-conditioned aged MSCs to secrete paracrine effectors, such as *IGF-1, FGF-2, SDF-1α* and *VEGF*. We measured the expression of these factors 7 and 30 days after transplantation and observed a significant increase in hearts transplanted with pre-conditioned aged MSCs. We and others have previously shown that paracrine signalling declines in MSCs with age [16, 36, 37] and therefore support our hypothesis to implement a pre-conditioning strategy that would aim to increase paracrine signalling in aged MSCs. Furthermore, our results demonstrate that treatment of aged MSCs with glucose depletion can increase paracrine signalling and may represent a feasible therapeutic option. These results concur to previous studies showing an increased paracrine signalling in MSCs pre-conditioned with different strategies [38–40].

Success of any pre-conditioning strategy depends considerably on the ability of conditioned cells to survive within infarcted myocardium. Our results demonstrated a decrease in percentage of apoptotic nuclei in myocardium transplanted with pre-conditioned aged MSCs compared with untreated cells. This was further supported by an observed up-regulation of *AKT, BCL-2* and *BCL-XL* at 7 and 30 days after transplantation and decrease in expression of pro-apoptotic genes *BAX* and *BAK*. A number of studies indicate that paracrine effect of *VEGF, IGF-1* and *FGF-2* can induce BCL2 expression in cardiomyocytes [41]. Gene expression of *MEF-2, NKX2.5* and *GATA-4* was assessed at day 7 and 30 after transplantation of MSCs into aged infarcted heart. *MEF-2* is involved in cardiac morphogenesis, whereas *GATA-4* and *NKX2.5* are early markers of pre-cardiac cells and are involved in heart formation. The expression of *MEF-2, NKX2.5* and *GATA-4* was higher in GsF-M transplanted MSCs group as compared with sham and NsF-M transplanted group.

MSC potential to repair senescent infarcted myocardium declines with age and concurs with similar reports showing impaired myocardial repair by age affected MSCs [2, 16]. Nevertheless, pre-conditioning of aged MSCs with glucose depleted medium can improve survival signalling, proliferation and retard senescent morphological changes. Moreover, transplantation of the pre-conditioned cells in senescent myocardium with infarction leads to improved repair as consequence of increased MSCs survival and paracrine signalling.

## Supporting information

**Fig. S1.** Expression analysis of AKT phosphorylation after pre-conditioning of aged MSCs. (**A**) Expression of pAKT/t-AKT. (**B**) Blot quantification, **P* < 0.05, ***P* < 0.01, ****P* < 0.001 for aged MSCs *versus* aged pre-conditioned MSCs.
