# Supplementary figures and images for: Mesenchymal stem cells conditioned with glucose depletion augments their ability to repair-infarcted myocardium
Source: J Cell Mol Med. 2012 Sep 26;16(10):2518–29. doi: 10.1111/j.1582-4934.2012.01568.x (PMC3823444; doi:10.1111/j.1582-4934.2012.01568.x)

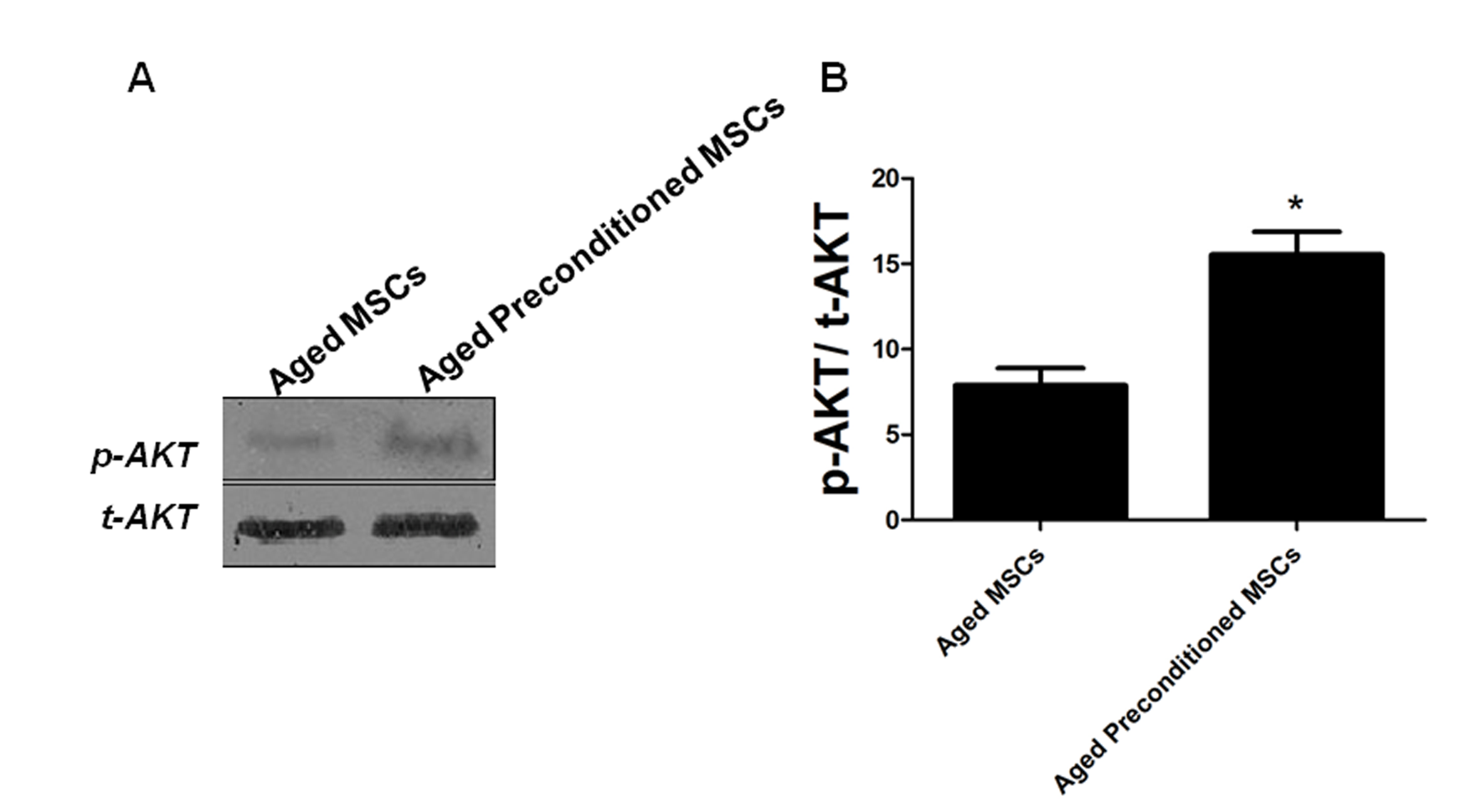

Supplement: Supplementary file 1 [file jcmm0016-2518-SD1.tif]
